# Prophylactic treatments for vestibular migraine: a systematic review and network meta-analysis of randomized clinical trials

**DOI:** 10.3389/fphar.2023.1332973

**Published:** 2023-12-21

**Authors:** Hongyuan Chu, Yuru Wang, Xia Ling, Kangzhi Li, Xu Yang

**Affiliations:** ^1^ Department of Neurology, Peking University Aerospace School of Clinical Medicine (Aerospace Center Hospital), Beijing, China; ^2^ Department of Pediatrics, Peking University First Hospital, Beijing, China; ^3^ Department of Neurology, Peking University First Hospital, Beijing, China

**Keywords:** vestibular migraine, prophylactic treatments, preventive treatment, valproate acid, flunarizine

## Abstract

**Objectives:** We compared and ranked the efficacy and tolerability of multiple prophylactic treatments for vestibular migraine (VM), including β-blockers, calcium channel blockers, antiseizure medications, and antidepressants such as tricyclics and serotonin–noradrenaline reuptake inhibitors.

**Methods:** PubMed, Web of Science, Embase, and Cochrane Center for Clinical Trials were systematically searched for relevant randomized clinical trials (RCTs) from March 2023 to May 2023. Studies on the efficacy and tolerability of prophylactic treatments for VM were included. Efficacy was measured using the average vertigo frequency per month and dizziness handicap inventory (DHI) improvement after 3–6 months of treatment. Tolerability was measured by the number of patients reporting at least one adverse event (AE). Network meta-analyses were performed according to a Bayesian framework and a random-effects model based on odds ratios or mean differences (MDs) and 95% confidence intervals (CIs). A sequence of ranking probability was calculated according to the surface under the cumulative ranking (SUCRA) curve. This network meta-analysis was previously registered with PROSPERO (CRD42023422258).

**Results:** Five RCTs comprising 334 patients were analyzed by synthesizing the published evidence. Considering the examined prophylactic therapies, there is significant evidence that valproate acid (VPA) is superior to placebo or abortive treatment alone (MD = −4.12, 95% CI = −8.09, −0.15) in reducing the frequency of vertigo. Flunarizine (MD = 20.00, 95% CI = 10.90, 29.10), valproate acid (MD = 18.88, 95% CI = 10.42, 27.34), and venlafaxine (MD = 11.48, 95% CI = 9.84, 13.12) were significantly more effective than placebo or abortive treatment in reducing DHI. VPA most strongly reduced the frequency of vertigo according to SUCRA, but it ranked third-to-last in tolerability. Flunarizine ranked best in DHI improvement but worst in tolerability. Metoprolol ranked worst for efficacy but best for tolerability.

**Conclusion:** VPA and flunarizine reduced the frequency of vertigo and improved DHI, but they had unfavorable tolerability. The effects of metoprolol on vertigo require further study. Given the low certainty and limited sample, additional head-to-head RCTs are warranted to further confirm efficacy.

**Systematic Review Registration:** https://www.crd.york.ac.uk/PROSPERO/; Identifier CRD42023422258.

## Introduction

Vestibular migraine (VM) is an underdiagnosed but increasingly recognized central condition that is characterized by recurrent vertigo attacks accompanied by migraine symptoms (Authors Anonymous, 2018). As the most common cause of episodic vertigo in both adults and children, VM affects up to 1.0%–2.7% of the population (Beh, 2022). Also previously known as “migrainous vertigo,” “migraine-associated vertigo,” “migraine-associated dizziness,” “migraine-anxiety-associated dizziness,” and “migraine-related vestibulopathy,” the term “vestibular migraine” has been accepted by the International Classification of Headaches as the unifying terminology that identifies both vestibular and migraine symptoms (Smyth et al., 2022).

The current consensus criteria for the diagnosis of VM were first published in 2012 by the International Bárány Society (Lempert et al., 2022). The current criteria are as follows: at least five episodes of vestibular symptoms (vertigo or dizziness) lasting between 5 min and 72 h, current or previous history of migraine with or without aura, one or more migraine features with at least 50% of the vestibular episodes, and not better explained by another diagnosis (Authors Anonymous, 2018).

The underlying pathophysiology of VM remains poorly understood, and most of the hypotheses are based on the established knowledge of migraine headaches. However, numerous theories have been formulated to explain the pathogenesis of VM, including cortical spreading depression, transmitter/vascular/inflammatory mechanisms, genetic predisposition, and functional brain changes (Furman et al., 2013). It has been suggested that brainstem vestibular nuclei and trigeminal nociceptive inputs are also involved in the pathogenesis of VM (Espinosa-Sanchez and Lopez-Escamez, 2015). Lastly, as some neurotransmitters (e.g., serotonin, noradrenaline, and dopamine) might be involved in the pathogenesis of VM, they could represent treatment options for this condition (Balaban, 2011).

Because current migraine treatment guidelines are based on migraines for which the ability of interventions to control vestibular symptoms was not assessed, there remains a clinical need for pragmatic management guidelines specific for VM using the available evidence.

Current pharmacological therapies can either be prophylactic to reduce the frequency and severity of future episodes or abortive to alleviate an acute attack. Because there are limited studies evaluating the efficacy of abortive treatments, this systematic review focuses on a comparison of prophylactic treatments.

There are various prophylactic treatment options addressing multiple aspects of VM, including β-blockers (such as propranolol and metoprolol), calcium channel blockers (such as flunarizine), antiseizure medications (such as topiramate and sodium valproate), antidepressants (such as venlafaxine), and emerging new drugs such as monoclonal antibodies against CGRP (such as erenumab, fremanezumab, and galcanezumab) (Smyth et al., 2022; Webster et al., 2023). As the mechanisms of action of these treatments are being studied and potential new targets are emerging, we can foresee a prospective future for the treatment of VM.

Because most previous studies comparing existing VM treatments on vestibular symptoms were observational, our systematic review was limited regarding the number of randomized clinical trials (RCTs) that compared these treatments. However, to the best of our knowledge, this is the first network meta-analysis (NMA) synthesizing established RCTs for VM and evaluating the efficacy and tolerability of multiple prophylactic treatments.

## Methods

This NMA was previously registered with PROSPERO (CRD42023422258), and it adhered to the PRISMA statement for network meta-analyses (Supplementary Material) (Hutton et al., 2015).

### Search strategy

Online databases including PubMed, Web of Science, Embase, and the Cochrane Center for Clinical Trials were systematically searched for relevant RCTs from 31 March 2023 to 1 May 2023. The search terms included contained VM (and previous names such as migraine-associated dizziness) and prophylactic treatments, including β-blockers, calcium channel blockers, antiseizure medications, vestibular rehabilitation, and antidepressants (the detailed search strings is presented in the Supplementary Material). The type of study was restricted to RCT. There was no limitation concerning year, publication, or language. Relevant reviews were retrieved, and their references were hand-searched.

### Study inclusion

Studies were independently selected by two reviewers (H.C. and Y.W.) by screening titles and abstracts according to previously formulated criteria. Differences in opinion were discussed to obtain consensus. If consensus could not be achieved, arbitration was provided by X.Y.

### Inclusion criteria

1) Patients: patients aged >18 with definite or probable VM could be included. Older studies containing diagnoses of “migrainous vertigo” or “migraine-associated vertigo” were also acceptable.
2) Interventions: prophylactic treatments for VM, including drugs, rehabilitation, and physical therapies, were considered eligible, regardless of whether they were combined with abortive treatments during vertigo episodes.
3) Outcomes: the efficacy outcome included either the average attack frequency per month after 3–6 months of treatment or symptomatic improvement as measured by questionnaires. The tolerability outcome included the number of patients reporting at least one adverse event (AE).

### Data extraction and risk of bias evaluation

Data were extracted on previously designed spreadsheets and rechecked by two reviewers (H.C. and Y.W.). For one study described in two or more articles, all available data were obtained to provide a comprehensive outcome. To evaluate the risk of bias of the included RCTs, the Cochrane Collaboration Risk of Bias tool version 2 (RoB2) was adopted (Sterne et al., 2019).

### Data synthesis

NMA was performed using R software (version 4.2.1, http://www.r-project.org). Specifically, the gemtc package used JAGS 4.3.0 based on a Bayesian framework, and the netmeta package was based on a frequentist framework (Turner et al., 2012; Cipriani et al., 2013). After extracting the outcomes as odds ratios (ORs) or mean differences (MDs) and 95% confidence intervals (CIs) for binary or continuous variables, respectively, we performed a random-effects meta-analysis. For included studies with more than one arm, each treatment arm was pooled to form a single node for the corresponding prophylactic treatment. Markov chain Monte Carlo methods were conducted for the prophylactic treatments to synthesize the results of direct and indirect comparisons (Lu and Ades, 2004). To quantify and evaluate heterogeneity, *I* ^*2*^ was calculated, wherein *I* ^*2*^ > 50% indicated high heterogeneity (Higgins et al., 2003). The surface under the cumulative ranking (SUCRA) curve of the ranking probability was calculated to evaluate the efficacy and tolerability of the investigated treatments. A higher SUCRA denotes superior tolerability, a longer latency between vertigo episodes, or improvement on the DHI.

## Results

### Description of included studies

**FIGURE 1:**
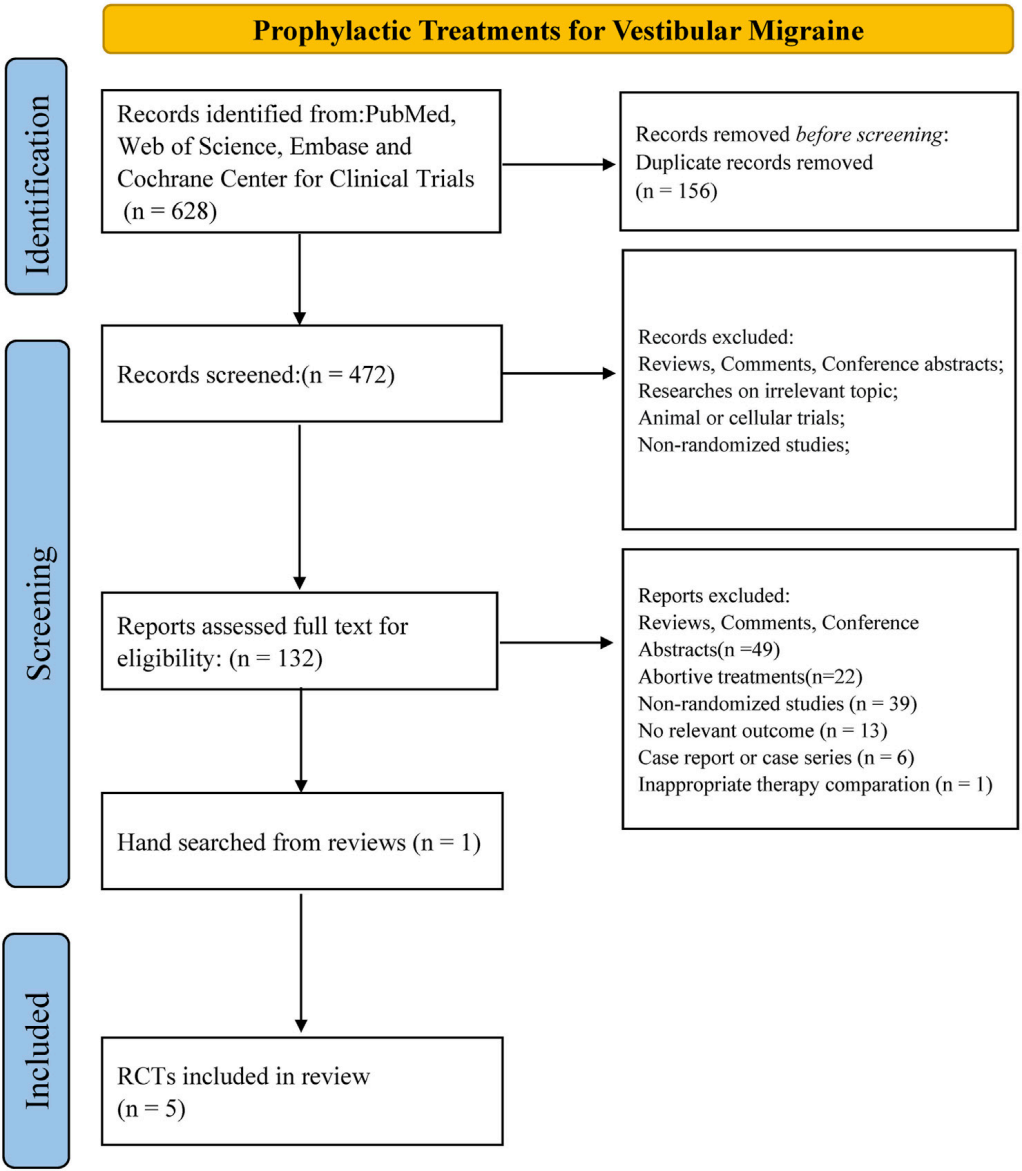
Flow chart of data retrieval.

In total, 628 results were identified from PubMed, Web of Science, Embase, and the Cochrane Center for Clinical Trials. We removed 156 duplicates and assessed 472 abstracts. After checking 132 full-text articles for eligibility, we excluded 128 articles. One article was identified via hand-search of the references of a relevant review. In total, five RCTs including 334 patients were analyzed in this NMA (Lepcha et al., 2014; Salviz et al., 2016; Yuan et al., 2016; Liu et al., 2017; Bayer et al., 2019; Li et al., 2021) (Figure 1).

Baseline information and demographic characteristics are presented in Tables 1–3. The proportions of patients with definite and probable VM, age, gender, study design, dose, and the durations of treatments were obtained. All five RCTs assessed the efficacy and tolerability of prophylactic treatments (abortive treatment during episodes alone or placebo, three arms; flunarizine, three arms; venlafaxine, two arms; VPA, one arm; propranolol, one arm; metoprolol, one arm). The risk of bias was evaluated using the RoB2 tool (Table 4). Heterogeneity was low among the included RCTs, as the *I* ^*2*^ in NMA analyses ranged between 4% and 12%.

**TABLE 1:** Characteristics of the included studies.

Participant information									
Study ID	Diagnosis	Number of participants per arm		Mean age		Female/Total (n)		Probable VM	Definite VM
		Arm 1	Arm 2	Arm 1	Arm 2	Arm 1	Arm 2		
Lepcha et al. (2014)	Migrainous vertigo	Betahistine and paracetamol (n = 26)	Flunarizine, betahistine and paracetamol (n = 26)	“Balanced”		18/26	16/26	N/A	N/A
Bayer et al. (2019)	VM	Placebo (n = 59)	Metoprolol (n = 62)	42.8 (14.3)	44.4 (14.2)	36/59	43/62	50	80
Liu et al. (2017)	VM	Venlafaxine (n = 23)	Flunarizine (n = 22)	53.22(15.5)	51.45(15.4)	16/23	14/22	45	20
		Valproic acid (n = 20)		52.35(16.0)		15/20			
Yuan et al. (2016)	VM	Betahistine (n = 12)	Flunarizine and betahistine (n = 13)	45.33(6.84)		27/32		0	32
Salviz et al. (2016)	VM	Venlafaxine (n = 31)	Propranolol (n = 33)	38	42	28/31	31/33	0	64
Studies involving definite VM only	VM	89		41.54		86/96		0	96
Total	VM	327		45.01		244/334		95	196

“N/A” represents not mentioned or not taken down for reasons. VM: vestibular migraine.

**TABLE 2:** Description of intervention and outcome in the included studies.

Intervention information					
Study ID	Description		Study duration	Vertigo severity and frequency change post treatment	
	Arm 1	Arm 2		Arm1	Arm2
Lepcha et al. (2014)	Betahistine16 mg thrice a day and paracetamol 1 g during episodes	Flunarizine 10 mg/d; betahistine16 mg thrice a day and paracetamol 1 g during episodes	12 weeks	“Marked improvement” in vertigo severity: 14/23; in headache severity: 10/23	“Marked improvement” in vertigo severity: 22/25; in headache severity: 19/25
Bayer et al. (2019)	Placebo	Metoprolol succinate 95 mg/day	6-month treatment+3-monoth follow-up	Average monthly vertigo attacks decreased from 4.5 to 3.1	Average monthly vertigo attacks decreased from 4.2 to 2.8
Liu et al. (2017)	37.5 mg/d venlafaxine (lower than normal dose)	10 mg/d flunarizine	3 months	Average monthly vertigo frequency decreased from 5.83 to 3.09	Average monthly vertigo frequency decreased from 4.95 to 4.15
				Average DHI decreased from 41.74 to 31.3	Average DHI decreased from 46.64 to 39.82
	1,000 mg/day valproic acid			Average monthly vertigo frequency decreased from 5.1 to 2.35	
				Average DHI decreased from 46.80 to 38.7	
Yuan et al. (2016)	Betahistine12 mg thrice a day during episodes	Flunarizine 10 mg/d; betahistine12 mg thrice a day during episodes	3 months	Average 3-month vertigo frequency decreased from 7.27 to 5.55	Average 3-month vertigo frequency decreased from 7.25 to 2.25
Salviz et al. (2016)	Propranolol at a flexible dose of 40 mg–160 mg	Venlafaxine 75 mg at bedtime	4 months	Average monthly vertigo frequency decreased from 12.6 to 1.9	Average monthly vertigo frequency decreased from 12.2 to 2.6
				Average DHI decreased from 55.8 to 31.3	Average DHI decreased from 50.9 to 19.9

“N/A” represents not mentioned or not taken down for reasons. DHI: dizziness handicap inventory.

**TABLE 3:** Design information of included studies.

Study design information				
Study ID	The use of multicenter design	Blinding	Registration	Patient enrollment
Lepcha et al. (2014)	In a tertiary academic referral center (no)	Not mentioned	N/A	Between July 2010 and August 2011
Bayer et al. (2019)	In tertiary referral centres (yes)	Yes	EudraCT, 2009-013701-34	Between June 2012 and April 2017
Liu et al. (2017)	Shandong Qianfoshan Hospital (no)	Single blind	ChiCTR-OPC-17011266	Between January 2016 and December 2016
Yuan et al. (2016)	Xinjiang People’s Hospital (no)	Single blind	N/A	Between July 2013 and May 2014
Salviz et al. (2016)	Haseki Training and Research Hospital (no)	Open-label	NCT02350985	Between 1 January 2014 and 15 September 2014

**TABLE 4:** Risk of bias assessments.

Study ID	Overall bias	Randomization process	Deviations from intended interventions	Missing outcome data	Outcome measurement	Report selection
Lepcha et al. (2014)	High risk	Some concerns	High risk	Some concerns	Low risk	Low risk
Bayer et al. (2019)	Low risk	Low risk	Low risk	Low risk	Low risk	Low risk
Liu et al. (2017)	Low risk	Some concerns	Low risk	Low risk	Low risk	Low risk
Yuan et al. (2016)	Low risk	Low risk	Low risk	Low risk	Low risk	Low risk
Salviz et al. (2016)	Low risk	Some concerns	Low risk	Low risk	Low risk	Low risk

### Efficacy outcomes

**FIGURE 2:**
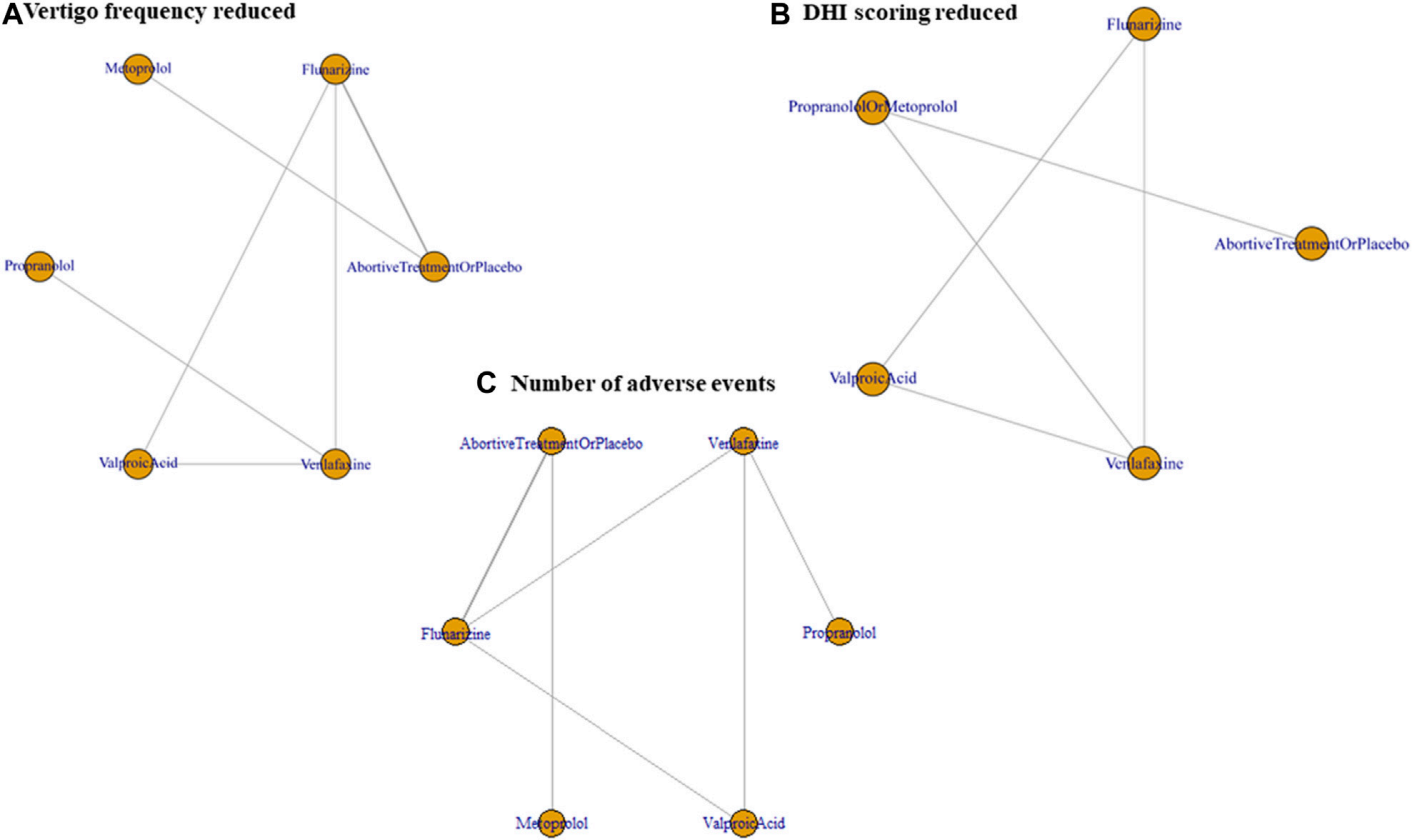
Network plot of prophylactic therapies comparing efficacy and tolerability. **(A)** Vertigo frequency reduction, **(B)** DHI improvement, and **(C)** the number of patients reporting any AE, all after 3–6 months of treatment. DHI, dizziness handicap inventory; AE, adverse event.

Outcomes regarding the improvement of DHI and frequency of vertigo after 3–6 months of treatment were analyzed. The intention-to-treat population was used instead of the per-protocol population in the included studies. The network plots presented in Figures 2A–C summarize the comparisons, and thicker lines indicate more head-to-head studies.

**FIGURE 3:**
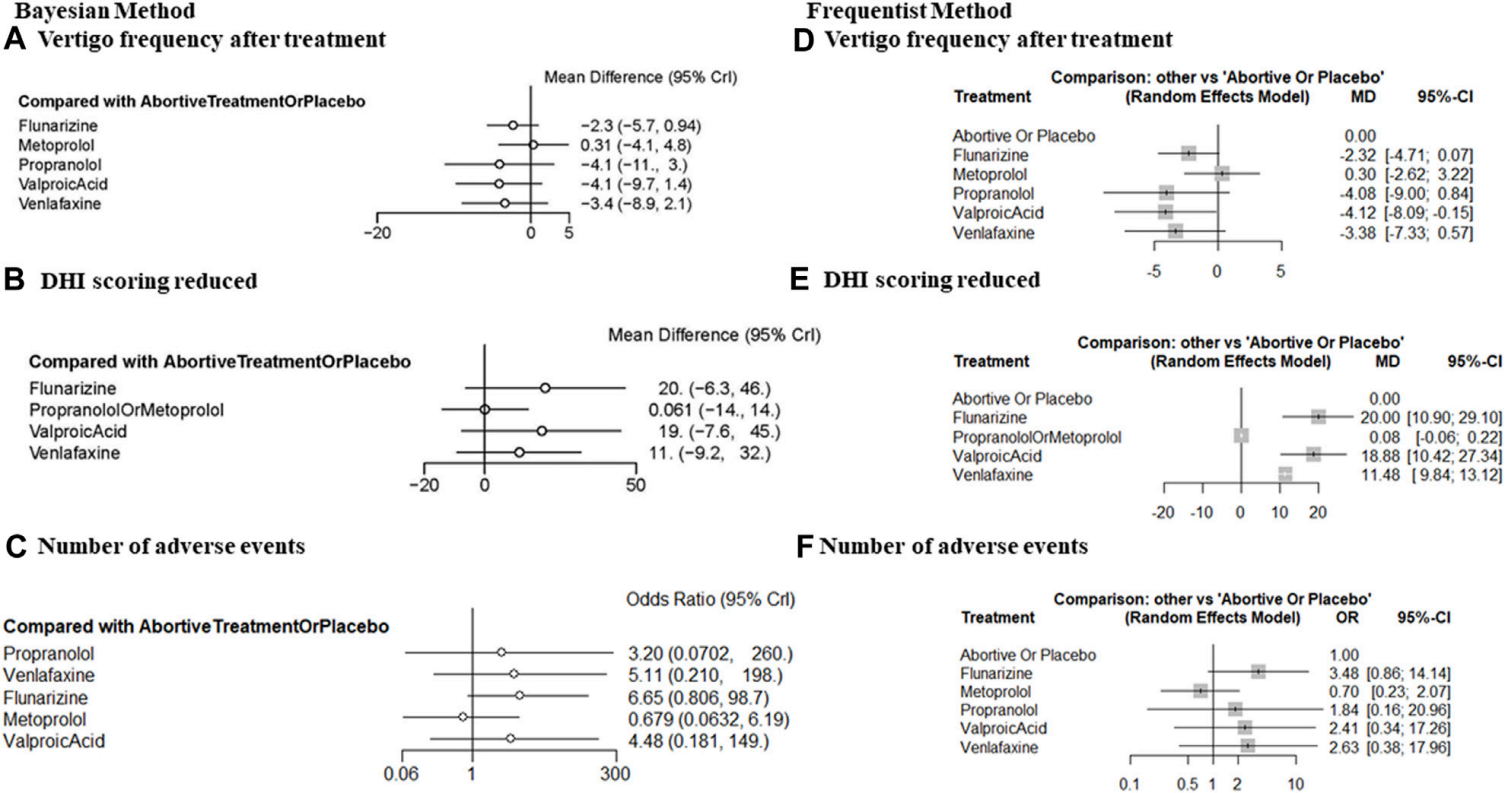
Forest plot comparing the efficacy and tolerability of prophylactic therapies with placebo or abortive treatment alone after 3–6 months of treatment. **(A)** The frequency of vertigo after treatment (Bayesian method), **(B)** DHI improvement (Bayesian method), **(C)** the number of patients reporting any AE, (Bayesian method); **(D)** the frequency of vertigo after treatment (frequentist method), **(E)** DHI improvement (frequentist method), and **(F)** the number of patients reporting any AE (frequentist method). DHI, dizziness handicap inventory; AE, adverse event.

The frequentist method forest plot illustrated that VPA (MD = −4.12, 95% CI = −8.09, −0.15) significantly reduced the frequency of vertigo versus abortive therapy alone or placebo (Figure 3D). The Bayesian method failed to detect any significant difference because of the broad 95% CI (Figure 3A).

Concerning DHI improvement after treatment, a significant effect of prophylactic therapy compared with abortive therapy alone or placebo was detected for flunarizine (MD = 20.00, 95% CI = 10.90, 29.10), valproate acid (MD = 18.88, 95% CI = 10.42, 27.34), and venlafaxine (MD = 11.48, 95% CI = 9.84, 13.12; Figure 3E). The β-blockers metoprolol and propranolol were pooled together to ensure the connection of the comparison network. For these β-blockers, there was no significant improvement (MD = 0.08, 95% CI = −0.06, 0.22). We interpret this finding with caution because the efficacy of metoprolol and propranolol could differ. Broad 95% CIs were observed in the Bayesian method because of the limited number of available RCTs and methodological limitations (Figure 3B).

**FIGURE 4:**
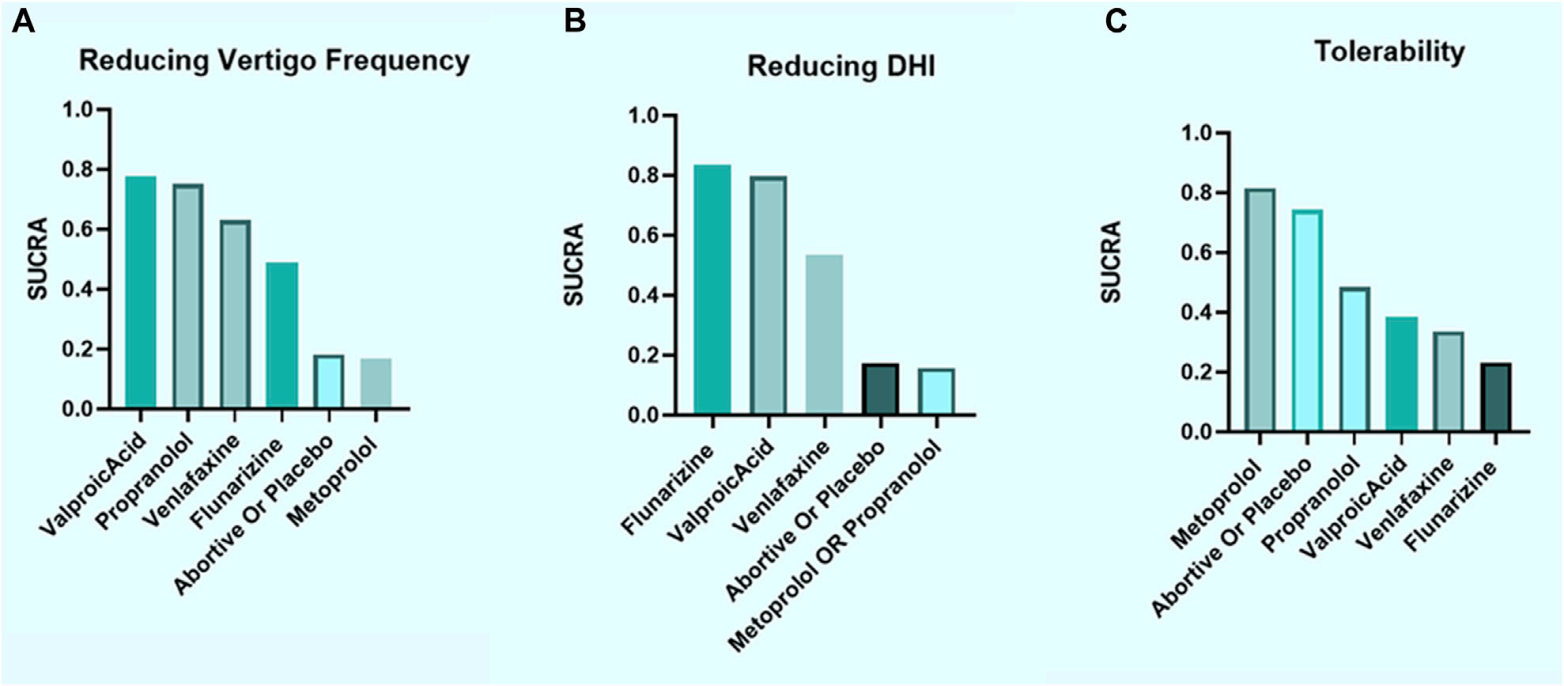
Ranking according to SUCRA for efficacy and tolerability. **(A)** Vertigo frequency reduction, **(B)** DHI improvement, and **(C)** tolerability considering the number of patients reporting any AE, all after 3–6 months of treatment. SUCRA, surface under the cumulative ranking curve; DHI, dizziness handicap inventory; AE, adverse event.

According to SUCRA, we established a ranking of efficacy for reducing the frequency of vertigo as follows: VPA > propranolol > venlafaxine > flunarizine > placebo or abortive treatment alone > metoprolol (Figure 4A). Regarding DHI improvement, the drugs were ranked in the order flunarizine > VPA > venlafaxine > placebo or abortive treatment alone > metoprolol or propranolol (Figure 4B). Most of the evaluated prophylactic treatments (excluding β-blockers) performed better than placebo or abortive treatment alone, indicating generally favorable efficacy against VM.

Although propranolol more effectively reduced the frequency of vertigo than metoprolol, there was no significant improvement in DHI with either treatment. However, given the low certainty of the results with the β-blockers because of the limited number of available RCTs, their efficacy in treating VM remains unclear. Therefore, further research with propranolol and metoprolol is warranted.

Although other data such as headache frequency, visual analog scales, vertigo symptom scales, and vestibular activities of daily living scales were also collected in our review, the limited number of RCTs that reported these outcomes prevented further analysis. Therefore, future studies addressing these outcomes are needed.

### Tolerability outcomes

Tolerability outcomes were measured as the number of patients reporting any AEs. No significantly increased risk of AEs was observed. This conclusion was firm in both Bayesian and frequentist frameworks (Figures 3C, F).

According to SUCRA, the tolerability of the treatments was ranked as follows: metoprolol > placebo or abortive treatment alone > propranolol > VPA > venlafaxine > flunarizine (Figure 4C). Although only measuring the number of AEs regardless of their types could resulted in misinterpretation, β-blockers displayed favorable tolerability.

## Discussion

In this network meta-analysis, we systematically summarized the comparative efficacy and tolerability of all available pharmacological interventions used as prophylactic treatments in patients with VM.

Most prophylactic therapies appeared to have good efficacy in reducing the frequency of vertigo and improving DHI. However, treatments with greater efficacy frequently had poor tolerability. VPA ranked first in reducing the frequency of vertigo but third-to-last in tolerability, whereas flunarizine ranked first in improving DHI but last in tolerability. Conversely, metoprolol, which ranked last in efficacy, ranked first in tolerability.

β-blockers have been commonly used for the prophylactic treatment of migraines (Smyth et al., 2022). Although the pathophysiology of migraines and the mechanisms of action of β-blockers in migraine prevention remain unclear, their high tolerability profile is encouraging for their clinical use. β-blockers might also provide some benefits concerning efficacy. Propranolol ranked second in reducing vertigo frequency, although it did not effectively improve DHI. This is in accordance with previous studies demonstrating that propranolol has high affinity for 5-hydroxytryptamine (serotonin or 5-HT) receptors, namely, 5HT2B and 5HT2C, which play a pivotal role in the pathophysiology of migraines. Propranolol also inhibits nitric oxide production by blocking inducible nitric oxide synthase, potentially suppressing the activation of the trigeminovascular complex (Fumagalli et al., 2020).

We advise caution in overinterpreting the inferior ranking of metoprolol regarding both the frequency of vertigo and DHI because the evidence was limited in the available RCTs. The only available RCT did not reach its target sample size because of early trial closure. Consequently, confirmatory analyses could not be performed. Furthermore, that trial had a relatively short duration, including a follow-up period of only 12 weeks. Therefore, additional studies with longer durations are necessary to obtain more comprehensive findings (Bayer et al., 2019). Together with propranolol, metoprolol is the only other β-blocker with the highest level of evidence and fewer side effects in major migraine guidelines. Pharmacologically, it is a moderate lipophilic β-1 selective antagonist, and unlike propranolol, it does not have affinity for 5-HT receptors (Fumagalli et al., 2020). In patients treated with β-blockers, the visual evoked potential amplitude tends to normalize, suggesting that β-blockers modulate cortical excitability and abnormal cortical information processing in migraines. Given the differences in the pharmacological mechanisms of metoprolol and propranolol, the pharmacological mechanisms of metoprolol might have less overlap with the possible pathogenesis of VM, which could explain why metoprolol is less effective than propranolol in the preventive treatment of VM in clinical practice.

VPA is an FDA-approved antiseizure medication for the preventative treatment of migraines (Khani et al., 2021). In our NMA, VPA ranked best in reducing the frequency of vertigo. This favorable efficacy might be associated with its mechanism. VPA increases GABA activity and inhibits NMDA-evoked neuroexcitatory signals, likely blocking cortical spreading depression during a migraine attack (Shnayder et al., 2023). VPA might also produce efficacy in patients with CACNA1A mutations by adjusting calcium channels (Espinosa-Sanchez and Lopez-Escamez, 2015; Hautakangas et al., 2022). However, tolerability must be considered when using VPA to treat VM based on its poor tolerability profile in this study. The AEs of VPA include asthenia/fatigue, dizziness/vertigo, nausea, tremor, and weight gain (Linde et al., 2013), as well as somnolence and dyspepsia (Liu et al., 2017).

Flunarizine is a non-selective calcium channel blocker that has been used to treat migraines since the 1980s (Louis, 1981). It has high lipid solubility, and it can cross the blood–brain barrier to antagonize histamine H1(Dyhrfjeld-Johnsen and Attali, 2019) and dopamine D2 receptors (Brücke et al., 1995). Our results illustrated that flunarizine has the best efficacy in improving DHI; however, it performed poorly in the vertigo frequency and tolerability outcomes. Flunarizine might increase various side effects such as extrapyramidal disturbances, somnolence, depression, weight gain, and drowsiness (Leone et al., 1991). In our study, the incidence of side effects of flunarizine in RCTs, including somnolence and weight gain (Lepcha et al., 2014), was as high as 24%. Some patients even withdrew from the study because of these side effects, and they were lost to follow-up (Li et al., 2021). In clinical applications, long-term administration of flunarizine should be carefully monitored.

Venlafaxine, which is a selective serotonin and noradrenaline reuptake inhibitor (Smyth et al., 2022), ranked third in reducing the frequency of vertigo, third in DHI improvement, and second-to-last in tolerability. The reported AEs of venlafaxine in the included RCTs were nausea, insomnia, palpitations, and somnolence (Liu et al., 2017). It should be noted that withdrawal syndrome can complicate the clinical use of venlafaxine (Smyth et al., 2022). Therefore, venlafaxine should be cautiously recommended for the preventive treatment of VM.

Together, prophylactic therapies exhibited good efficacy in reducing the frequency of vertigo and improving DHI. Respective strengths were observed among the drugs regarding the frequency of vertigo, DHI improvement, and tolerability.

## Limitation

The limited number of RCTs restricted further analyses of important outcomes such as headache frequency, visual analog scales, vertigo symptom scales, and quality of life. Further, although we adopted a statistically suitable and appropriate NMA methodology, significance might have been underestimated. Although heterogeneity was low based on the *I* ^*2*^ statistic, there could be incompatibilities in the baseline characteristics among the arms of the included RCTs. Both “probable” and “definite” VM were included in this review (although including only definite VM would largely strengthen the evidence, which was prevented in this review because of the limited number of studies that implemented this restriction). Hence, we strongly recommend that future researchers enroll only patients with definite VM. More RCTs are needed to clarify the efficacy and tolerability of prophylactic therapies for VM.

## Conclusion

VPA and flunarizine appeared most effective in reducing the frequency of vertigo and improving DHI, but their tolerability was unfavorable. Conversely, metoprolol ranked last in efficacy for both the frequency of vertigo and DHI, but it ranked first in tolerability. This might emphasize the need for precision medicine in patients with different needs and symptoms. However, because of the limited number of available RCTs, additional RCTs comparing the efficacy and tolerability of prophylactic treatment for VM are warranted to confirm our findings.

## Data Availability

The original contributions presented in the study are included in the article/Supplementary Material, further inquiries can be directed to the corresponding author.
